# Antiproteinuric and Hyperkalemic Mechanisms Activated by Dual Versus Single Blockade of the RAS in Renovascular Hypertensive Rats

**DOI:** 10.3389/fphys.2021.656460

**Published:** 2021-06-09

**Authors:** José Wilson N. Corrêa, Karoline R. Boaro, Letícia B. Sene, Juliano Z. Polidoro, Thiago A. Salles, Flavia L. Martins, Lusiane M. Bendhack, Adriana C. C. Girardi

**Affiliations:** ^1^Laboratory of Genetics and Molecular Cardiology, Heart Institute (InCor) University of São Paulo Medical School, São Paulo, Brazil; ^2^Department of Physiological Sciences, Institute of Biological Sciences, Federal University of Amazonas, Manaus, Brazil; ^3^Faculty of Pharmaceutical Sciences of Ribeirão Preto, University of São Paulo, Ribeirão Preto, Brazil

**Keywords:** proteinuria, hypertension, podocin, cubilin, ClC-5 chloride channel, epithelial sodium channel, hyperkalemia, angiotensin II

## Abstract

This study aimed to investigate the antiproteinuric and hyperkalemic mechanisms activated by dual renin-angiotensin system (RAS) blockade in renovascular hypertensive rats (2-kidney 1-clip model [2K-1C]). Six weeks after clipping the left renal artery or sham operation (2K), rats were treated with losartan, enalapril, or both drugs for two weeks. We found that 2K-1C rats displayed higher tail-cuff blood pressure (BP), increased non-clipped kidney Ang II concentration, and more pronounced urinary albumin excretion than 2K. BP was decreased by the treatment with either enalapril or losartan, and the combination of both drugs promoted an additional antihypertensive effect in 2K-1C rats. Renal Ang II content and albuminuria were reduced by either enalapril or losartan in monotherapy and restored to control levels by dual RAS blockade. Albuminuria in 2K-1C rats was accompanied by downregulation of the glomerular slit protein podocin, reduction of the endocytic receptors megalin and cubilin, and a marked decrease in the expression of the ClC-5 chloride channel, compared to 2K animals. Treatment with losartan and enalapril in monotherapy or combination increased the expression of podocin, cubilin, and ClC-5. However, only the combined therapy normalized podocin, cubilin, and ClC-5 protein abundance in the non-clipped kidney of 2K-1C rats. Renovascular hypertensive 2K-1C rats had a lower concentration of plasma potassium compared to 2K rats. Single RAS blockade normalized potassium plasma concentration, whereas 2K-1C rats treated with dual RAS blockade exhibited hyperkalemia. Hypokalemia in 2K-1C rats was accompanied by an increase in the cleaved activated forms of α-ENaC and γ-ENaC and the expression of β-ENaC. Combined RAS blockade but not monotherapy significantly reduced the expression of these ENaC subunits in 2K-1C rats. Indeed, double RAS blockade reduced the abundance of cleaved-α-ENaC to levels lower than those of 2K rats. Collectively, these results demonstrate that the antiproteinuric effect of dual RAS blockade in 2K-1C rats is associated with the restored abundance of podocin and cubilin, and ClC-5. Moreover, double RAS blockade-induced hyperkalemia may be due, at least partially, to an exaggerated downregulation of cleaved α-ENaC in the non-clipped kidney of renovascular hypertensive rats.

## Introduction

The renin-angiotensin system (RAS) plays a crucial role in blood pressure (BP) control. However, abnormal stimulation of RAS components, ultimately leading to the upregulation of angiotensin II (Ang II) and activation of its angiotensin receptor type 1 (AT1R), contribute to the development and progression of hypertension (Crowley et al., 2005, 2006). Not surprisingly, pharmacological antagonists of the RAS, such as angiotensin-converting enzyme inhibitors (ACEi) or AT1R blockers (ARBs), constitute classical therapeutic approaches to control hypertension and end-organ damage, including the kidney (Carson et al., 2001; Dahlof et al., 2002; Viazzi et al., 2016). Interestingly, because of the “escape phenomenon” (Athyros et al., 2007), ACEi and ARB alone may not be sufficient to fully block the RAS. Indeed, evidence suggests that the combined use of ACEi and ARBs is superior to monotherapy for BP control and proteinuria reduction (Kunz et al., 2008; Mann et al., 2008). Nevertheless, it may also be associated with increased adverse effects (Doulton et al., 2005; Mann et al., 2008).

Proteinuria is a well-documented risk factor and predictor of progression to end-stage renal disease and cardiovascular disease (Lassila et al., 2004). The regulation of the expression of key proteins of the glomerular filtration barrier and the apical endocytic machinery in the renal proximal tubule is impaired in hypertension, producing increased urinary protein excretion (Tanaka and Nakaki, 2004; Inoue et al., 2013; Arruda-Junior et al., 2014; Berger et al., 2015; Seccia et al., 2017; Lopes et al., 2020). Lesions in the glomerular slit diaphragm, an intercellular junction between podocytes’ foot processes, are particularly important for establishing proteinuria (Wharram et al., 2005; Grahammer et al., 2013). Accordingly, renovascular-hypertensive rats exhibit increased proteinuria compared to normotensive rats, which is associated with downregulation of the podocin expression (Lopes et al., 2020). RAS status also seems important for glomerular integrity fate, as evidenced by studies with rats receiving Ang II chronic pressor doses. These animals presented podocyte apoptosis, reduction of nephrin protein abundance, and, as a result, proteinuria (Jia et al., 2008). Nonetheless, proteinuria in hypertensive models such as the spontaneously hypertensive rat (SHR) appears to be, at least in part, caused by a dysregulation of the proximal tubule endocytic machinery (Tanaka and Nakaki, 2004; Inoue et al., 2013). This macromolecular complex, comprised of megalin, cubilin, and the chloride channel ClC-5 (Gekle, 2005; Birn and Christensen, 2006; Hryciw et al., 2006; Pollock and Poronnik, 2007; Nielsen et al., 2016), among other components, is responsible for internalizing low molecular weight proteins capable of passing through the slit diaphragm. In this regard, SHRs exhibit lower expression of cubilin, megalin, ClC-5, and vH^+^-ATPase B2 subunit in the renal cortex compared to age-matched normotensive rats (Inoue et al., 2013). RAS upregulation might be important in the pathophysiology of proximal tubular proteinuria beyond high BP since the treatment of SHRs with losartan versus hydralazine produced upregulation of megalin expression in the renal proximal tubule, an effect that was accompanied by a much greater reduction in proteinuria. However, both antihypertensives displayed similar BP effects (Arruda-Junior et al., 2014). Despite the evidence of RAS modulating glomerular and proximal tubule handling of proteins, the molecular mechanisms that mediate the additive antiproteinuric effects of dual versus single blockade of the RAS remain to be established.

Clinical studies designed to evaluate the effects of the combination of ACEi and ARBs revealed additional side effects such as hyperkalemia and hypotension (Pfeffer et al., 2003; Phillips et al., 2007; Saglimbene et al., 2018), so there is a current contraindication for using dual RAS blockade in the management of hypertension. However, in terms of kidney disease prognosis, existing data regarding dual RAS blockade inhibition is still a matter of debate. Indeed, the result of a current meta-analysis demonstrates that dual RAS blockade may be suitable for hypertensive patients with diabetic kidney nephropathy and albuminuria (Feng et al., 2019). Noteworthy, the mechanisms by which the double combination of ARBs and ACEi may cause hyperkalemia have not been fully elucidated. The main proposed mechanisms include decreased aldosterone concentrations, reduced glomerular filtration rate, and/or abnormal collecting duct function (Raebel, 2012).

In light of the above, the present study aimed to investigate the underlying molecular mechanisms associated with the additive antiproteinuric and hyperkalemic effects activated by dual versus single blockade of the RAS in an experimental model of hypertension. We hypothesized that the dual blockade of RAS could better preserve both glomerular and proximal tubular components responsible for protein handling than monotherapy and that the increased risk of hyperkalemia could be established by exaggerated downregulation of sodium epithelial channel (ENaC) in collecting ducts.

## Materials and Methods

### Animal Protocols, Surgical Procedures, and Drug Treatment

**FIGURE 1:**
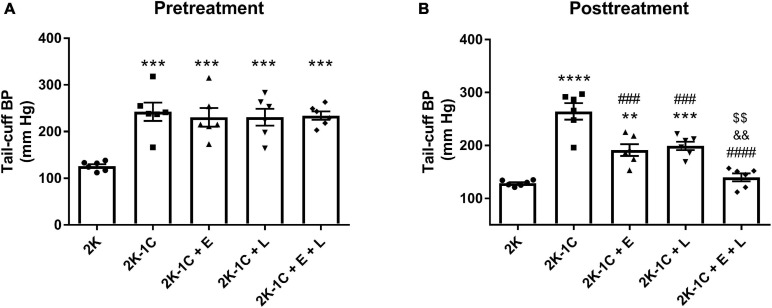
Dual RAS blockade, but not losartan or enalapril alone, normalizes blood pressure in renovascular hypertensive rats. Blood pressure was measured using tail-cuff plethysmography in two-kidney one-clip (2K-1C) renovascular-hypertensive rats and controls (2K). Experiments were conducted **(A)** before and **(B)** 14 days after treatment with enalapril (2K-1C + E, 20 mg/kg/day), losartan (2K-1C + L, 30 mg/kg/day), enalapril plus losartan (2K-1C + E + L, 30 and 20 mg/kg/day, respectively), or vehicle (2K and 2K-1C, water: 1 ml/kg). The values represent individual measurements and the means ± SEM. **P < 0.01, ***P < 0.001, and ****P < 0.0001 vs. 2K; ^###^*P* < 0.001 and ^####^*P* < 0.0001 vs. 2K-1C; ^&&^*P* < 0.01 vs. 2K-1C + E; ^$$^*P* < 0.01 vs. 2K-1C + L.

All animal procedures were approved by the Institutional Animal Care and Use Committee of the University of São Paulo, Ribeirão Preto, SP, Brazil (Protocol n° 07.1.607.53.1) and were carried out following the ethical principles of the Brazilian College of Animal Experimentation. Experiments were performed on male Wistar rats (8 weeks old, 180–200 g) purchased from the University of São Paulo (São Paulo, SP, Brazil). The animals were housed at the Heart Institute animal facility under a constant temperature and a 12:12-h dark-light cycle and had free access to food and water. The two-kidney one-clip (2K-1C) renovascular-hypertension was induced as described previously (Corrêa et al., 2020). Briefly, rats were anesthetized with tribromoethanol (50 mg/kg i.p.) and, after a midline laparotomy, a silver clip with an internal diameter of 0.20 mm was placed around the left renal artery. Normotensive two-kidney (2K) rats were subjected to the same surgical procedure except for the clip implantation. Tail-cuff BP was measured 6 weeks after surgery (pretreatment measurement) by plethysmography (BP-2000 Blood Pressure Analysis System, Visitech System, Apex, NC, United States), as previously described (Martins et al., 2020). Only 2K−1C rats that displayed tail-cuff BP higher than 160 mm Hg were included in the study (Figure 1A). The 2K−1C rats were then randomly divided into four groups and treated for 14 days with vehicle (water, 1 ml/kg), enalapril (20 mg/kg/day), losartan (30 mg/kg/day), or enalapril (20 mg/kg/day) plus losartan (30 mg/kg/day) by gavage. Vehicle-treated 2K rats were used as controls. Tail-cuff BP was also measured at the end of the treatment. Rats were anesthetized with ketamine and xylazine (50 and 10 mg/kg, respectively, i.p.). Blood samples were collected from the abdominal aorta and transferred into vacutainer tubes to obtain plasma. Subsequently, rats were euthanized by cervical displacement, and the kidneys were immediately removed and weighed. The left and right kidney weights were normalized by their respective tibial length. The non-clipped kidney was used to isolate renal cortical membrane and total proteins or tissue fixation for histological analysis.

### Renal Function Evaluation

The rats were individually housed and placed in metabolic cages (Tecniplast, Buguggiate, VA, Italy) throughout the last 4 days of treatment, as previously described (Inoue et al., 2013). Urine samples were collected for 24 h and used to determine urinary flow, sodium, potassium, glucose, creatinine, proteinuria, and albuminuria. Creatinine clearance was used to estimate the glomerular filtration rate (GFR).

### Plasma and Urine Analysis

Plasma and urinary sodium and potassium concentrations were measured by flame photometry (Digimed DM-62, São Paulo, Brazil). Plasma and urinary creatinine concentrations were measured using a kinetic method (Labtest, Lagoa Santa, MG, Brazil) and a ThermoPlate Analyzer Plus (ThermoPlate, São Paulo, SP, Brazil). The urinary glucose concentration was measured by the hexokinase method using a commercial kit (Labtest). Total urinary protein excretion was measured using a commercial kit based on the pyrogallol red-molybdate method (Labtest). The urinary albumin concentration was determined using an enzyme-linked immunosorbent assay (ELISA) kit specific for rat urine albumin (Nephrat II kit; Ethos Biosciences, Newtown Square, PA, United States). The experiments were carried out following the manufacturer’s instructions.

### Preparation of Renal Homogenate and Cortical Membrane Proteins

Kidney cortices were isolated at 4°C and homogenized in phosphate buffer saline (PBS) containing protease [1 mM pepstatin, 1 mM leupeptin, 230 mM PMSF and 1 tablet/50 ml of Complete protease inhibitor cocktail tablets (Roche, Mannheim, Germany)] and phosphatase inhibitors (50 mM NaF and 15 mM sodium pyrophosphate) at pH 7.4. Aliquots of the homogenate were saved for ELISA. The remaining renal cortical homogenate was centrifuged at 4,000 rpm for 15 min at 4°C. The supernatant was removed and subjected to a further 90 min centrifugation at 28,000 rpm at 4°C to pellet the membrane fraction. The supernatant was discarded; the renal cortical membranes were resuspended in fresh PBS containing protease and phosphatase inhibitors. The total protein concentration was determined using the Lowry method (Lowry et al., 1951).

### SDS-PAGE and Immunoblotting

Renal cortical samples containing an equivalent amount of protein and volumes of urine containing 5 μg of creatinine were solubilized in Laemmli sample buffer and resolved using 7.5 or 10% SDS-PAGE gels, respectively. Following electrophoresis, gels containing proteins from urine samples were silver stained using the ProteoSilver Plus kit (Merck, Darmstadt, Germany) to detect urinary proteins. Renal homogenate and urine proteins were transferred from SDS-PAGE gels to polyvinylidene difluoride (PVDF) membranes (Immobilon-P; Merck). Then, the PVDF membranes were incubated with blocking solution (5% non-fat dry milk or 5% bovine serum albumin and 0.1% Tween 20 in PBS, pH 7.4) for 1 h and the following specific primary antibodies overnight (4°C): polyclonal antibody against podocin (1:1,000; Santa Cruz Biotech, sc-21009), polyclonal antibody against nephrin (1:1,000; Abcam, Cambridge, MA, Ab 58968), polyclonal antibody against megalin (1:50,000; a gift from Dr. Daniel Biemesderfer) (Li et al., 2008), polyclonal antibody against cubilin (1:1,000; Santa Cruz Biotech, sc-20609), polyclonal antibody against the ClC-5, H^+^/Cl^–^ exchange transporter 5 (1:1,000; Alpha Diagnostic International, CLC51-A, San Antonio, TX, United States) (Carraro-Lacroix et al., 2010), polyclonal antibody against α-ENaC (1:1,000; Alomone Labs, ASC-030, Jerusalem, Israel), polyclonal antibody against β-ENaC (1:1,000; StressMarq Biosciences, SPC-404, Victoria, British Columbia, Canada), polyclonal antibody against cleaved γ-ENaC (1:1,000; Alomone Labs, ASC-011), or actin (1:5,000; Abcam ab179467). Actin was used as a loading control. Proteins were detected using horseradish peroxidase-conjugated secondary antibodies (1:2,000; Jackson ImmunoResearch, West Grove, PA, United States). The bound antibodies were detected using an enhanced chemiluminescence system (GE HealthCare, Chicago, IL, United States) according to the manufacturer’s protocols. The visualized bands were digitized using an image scanner (GE HealthCare) and quantified using Scion Image software (Scion Corporation, Frederick, MD, United States).

### Histological Analysis

The non-clipped kidney from five rats per group was fixed in 10% phosphate buffered-formalin, pH 7.3, for 24 h. After the fixation, the renal tissue was transferred into 70% vol/vol ethanol and stored at room temperature until processing into paraffin. The paraffin blocks were sectioned at 4 μm thickness using a microtome (Leica RM 2035, Leica Biosystems, Wetzlar, Hesse, Germany) and placed on silanized slides. Then, the sections were stained with Picrosirius red to detect collagen fiber deposition. Fifteen fields from each section were chosen at random, and the images were acquired under a 400× magnification light microscope using the Leica Qwin Plus Software (Leica Biosystems). The red-stained collagen fibers were quantified by Image J software, and the degree of fibrosis was expressed as the average percentage of the total area. A single examiner, blinded to the experimental groups, performed all histological measures.

### Determination of Renal Cortical Ang II and TGF-β and Plasma Aldosterone Concentrations

The Ang II and TGF-β concentrations in renal homogenates and the plasma aldosterone concentration were measured using competitive ELISA (Biomatik Corporation, Kitchener, ON, Canada), (Abcam), and (Enzo Life Sciences, New York, NY, United States), respectively, according to the manufacturers’ instructions, including sample collection and storage.

### Statistical Analysis

The results are reported as the mean ± standard error of the mean (SEM). Comparisons among the groups were performed using one-way ANOVA followed by the Tukey *post hoc* test. *P* < 0.05 was considered significant.

## Results

### Effect of Dual Versus Single RAS Blockade on Biometric Parameters, Blood Pressure, Renal Function, and Non-Clipped Kidney Ang II Concentration

The biometric characteristics of the animals are shown in Table 1. The final mean body weights were similar among the five groups of rats. Vehicle-treated 2K-1C rats displayed higher right kidney (non-clipped) weight to tibia length (TL) than normotensive 2K rats. In contrast, enalapril and losartan alone or in combination prevented non-clipped kidney hypertrophy. The left kidney (clipped) weight (LKW) to TL did not significantly vary among the five groups of rats, despite a trend toward a reduction in LKW/TL in vehicle-treated and drug-treated 2K-1C.

**TABLE 1:** Biometrical and renal function parameters, non-clipped kidney Ang II content and plasma aldosterone concentration in two-kidney one-clip (2K-1C) renovascular-hypertensive rats and controls (2K).

	2K (*n* = 6)	2K-1C (*n* = 6)	2K-1C + E (*n* = 6)	2K-1C + L (*n* = 6)	2K-1C + E + L (*n* = 6)
Body weight (g)	430 ± 9	376 ± 13	386 ± 22	375 ± 21	388 ± 20
LKW/TL (mg/mm)	0.40 ± 0.01	0.29 ± 0.04	0.33 ± 0.03	0.34 ± 0.03	0.29 ± 0.02
RKW/TL (mg/mm)	0.41 ± 0.01	0.61 ± 0.04**	0.53 ± 0.04	0.54 ± 0.04	0.44 ± 0.04
Urinary flow (ml/24h/kg)	35 ± 1	194 ± 19***	111 ± 18*^##^	118 ± 18*^#^	63 ± 9^###^
Plasma Cr (mg/dl)	0.43 ± 0.01	0.56 ± 0.03	0.59 ± 0.03	0.52 ± 0.07	0.53 ± 0.04
Cr Clearance (ml/min/kg)	9.1 ± 0.6	5.9 ± 0.5**	6.4 ± 0.5**	6.0 ± 0.3**	6.6 ± 0.3**
Plasma Na^+^ (mM)	141 ± 1	144 ± 3	143 ± 2	143 ± 3	142 ± 1
FE Na^+^ (%)	0.51 ± 0.04	0.72 ± 0.13	0.89 ± 0.02	0.87 ± 0.04	0.64 ± 0.11
Glycosuria (mg/24h/kg)	21 ± 1	16 ± 2	279 ± 28***^###^	200 ± 22***^###^	217 ± 16***^###^
Non-clipped kidney Ang II (pg/g)	133 ± 6	1422 ± 93***	337 ± 17*^###^	422 ± 22***^###^	93 ± 8^###,&&,[*dollar*][*dollar*][*dollar*]^
Plasma Aldosterone (pg/ml)	224 ± 14	881 ± 26***	445 ± 87^##^	489 ± 74^#^	310 ± 89^###^

Values are means ± SEM with the number of rats of each group in parenthesis. *P < 0.05, **P < 0.01, and ***P < 0.01 vs. 2K; ^#^*P* < 0.05, ^##^*P* < 0.01, and ^###^*P* < 0.001 vs. 2K-1C; ^&&^*P* < 0.01 vs. 2K-1C + E, ^$$$^*P* < 0.001 vs. 2K-1C + L, assessed by ANOVA one-way followed by Tukey’s *post hoc* test. Urinary output was measured gravimetrically. Fractional excretion (FE) of Na^+^ was expressed in percentage and calculated as C_*Na*_/C_*Cr*_ where C_*Na*_ is the Na^+^ clearance, and C_*Cr*_ is creatinine clearance. Ang II, angiotensin II; Cr, creatinine; LKW, left kidney weight; RKW, right kidney weight; TL, tibia length.

As shown in Figure 1A, the tail-cuff BP of 2K-1C rats was higher than that observed in 2K rats (242 ± 20 mmHg vs. 126 ± 4 mm Hg, *P* < 0.001). Before starting treatments, animals randomized to enalapril and losartan alone or in combination exhibited tail-cuff BP levels similar to that of 2K-1C rats (Figure 1A). After 14 days of treatment (Figure 1B), the BP levels of 2K-1C rats remained remarkably high compared to 2K rats (264 ± 15 mm Hg vs. 128 ± 2 mm Hg, *P* < 0.0001). Treatment with enalapril (191 ± 11 mm Hg) or losartan (199 ± 8 mm Hg) promoted a significant reduction in BP of 2K-1C rats (*P* < 0.01 and *P* < 0.001 vs. 2K-1C, respectively). In addition, the combination of enalapril and losartan exerted an antihypertensive effect, bringing the tail-cuff BP of double blockaded treated 2K-1C rats to levels non-significantly different from normotensive 2K control rats (140 ± 7 vs. 128 ± 2 mm Hg).

Vehicle-treated 2K-1C rats displayed higher urinary flow than 2K rats (Table 1). A reduction in urinary flow was observed in 2K-1C treated with enalapril or losartan. The enalapril/losartan combination promoted an additional decrease in 2K-1C urinary flow. Vehicle-treated 2K-1C rats also exhibited lower creatinine clearance than normotensive 2K rats. None of the treatments with ACEi and ARB alone or in combination could prevent/revert the decline in creatinine clearance (Table 1). 2K-1C rats with or without treatments did not show differences in plasma levels of creatinine and sodium and on fractional sodium excretion compared to 2K rats. Although 2K-1C rats did not show differences in urinary glucose excretion than 2K rats, it was observed that enalapril and losartan alone or in combination similarly increased glycosuria in 2K-1C hypertensive rats.

In addition, 2K-1C renovascular hypertensive rats showed a marked increase in Ang II content in the non-clipped kidney over 2K rats (Table 1). The treatment with losartan or enalapril reduced non-clipped kidney Ang II levels. The reduction of renal Ang II concentration was even more prominent when enalapril and losartan were used in combination.

### Effect of Dual Versus Single RAS Blockade on Urinary Protein and Albumin Excretion

**FIGURE 2:**
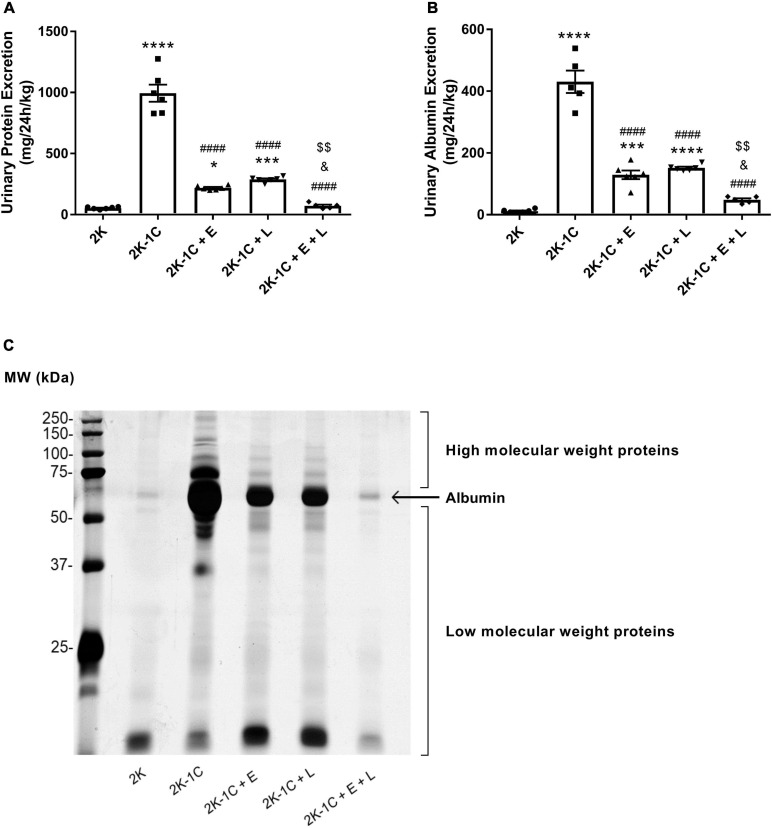
Dual RAS blockade, but not losartan or enalapril alone, normalizes proteinuria and albuminuria in renovascular hypertensive rats. Two-kidney one-clip (2K-1C) renovascular-hypertensive rats treated with enalapril (2K-1C + E, 20 mg/kg/day), losartan (2K-1C + L, 30 mg/kg/day), enalapril plus losartan (2K-1C + E + L, 30 and 20 mg/kg/day, respectively), or vehicle (2K-1C) and vehicle-treated control rats (2K) were placed into metabolic cages for 24-h urine collection. **(A)** Urinary protein excretion was measured using a commercially available kit based on the pyrogallol red-molybdate method. **(B)** Urinary albumin concentration was determined by ELISA. The values represent individual measurements and the means ± SEM. *P < 0.05,***P < 0.001, and ****P < 0.0001 vs. 2K; ^####^*P* < 0.0001 vs. 2K-1C; ^&^*P* < 0.05 vs. 2K-1C + E; ^$$^*P* < 0.01 vs. 2K-1C + L. **(C)** Profile of urine proteins excreted by the five groups of rats. The 24-h urine samples (volume equivalent to 5.0 μg of creatinine) were subjected to 10% SDS-PAGE. Following electrophoresis, the gels were silver stained. The urinary excretion of proteins of a size smaller than albumin (low molecular weight proteins) and higher than albumin (high molecular weight proteins) is indicated in the representative gel.

The urine analysis revealed that 2K-1C rats presented remarkably higher proteinuria (995 ± 70 vs. 54 ± 5 mg/24 h/Kg, *P* < 0.0001) (Figure 2A) and albuminuria levels (431 ± 36 vs. 12 ± 2 mg/24 h/kg, *P* < 0.0001) (Figure 2B) than 2K rats. The treatment with enalapril or losartan reduced both total protein excretion (enalapril: 219 ± 7 mg/24 h/kg, *P* < 0.0001; losartan: 287 ± 72 mg/24 h/kg, *P* < 0.0001) and albuminuria (enalapril: 129 ± 14 mg/24 h/kg, *P* < 0.0001; losartan:152 ± 4 mg/24 h/Kg, *P* < 0.0001), respectively, in 2K-1C rats. An additional reduction in proteinuria (72 ± 10 mg/24 h/kg) and albuminuria (48 ± 5 mg/24 h/kg) excretion were observed after treating 2K-1C rats with the combination of enalapril and losartan in comparison with monotherapy (*P* < 0.05) (Figures 2A,B). Dual RAS blockade restored proteinuria and albuminuria in 2K-1C to the same levels of normotensive 2K rats (Figures 2A,B). The profile of urinary proteins excreted by the five groups of rats was evaluated by SDS-PAGE (Figure 2C). Analysis of the pattern of proteins excreted by vehicle-treated 2K-1C rats suggested proteinuria of mixed origin, i.e., glomerular and tubular proteinuria, since both high- and low-molecular-weight proteins were present in the urine of these animals (Waller et al., 1989). The treatment of 2K-1C rats with enalapril or losartan reduced the protein excretion of high and low molecular weight. A much greater reduction was observed in the group of hypertensive 2K-1C rats that received the combined treatment. The results from these experiments are consistent with those shown in Figures 2A,B.

### Effect of Dual Versus Single RAS Blockade on the Expression of Components of the Glomerular Filtration Barrier

**FIGURE 3:**
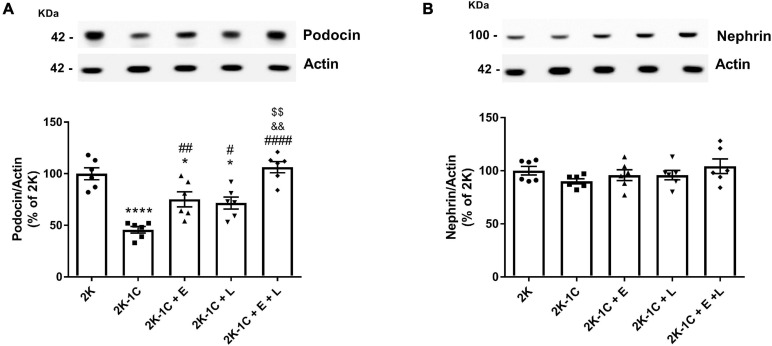
Effect of dual versus single RAS blockade on the expression of components of the glomerular filtration barrier in the non-clipped kidney of renovascular hypertensive rats. Samples isolated from the non-clipped kidney of renovascular-hypertensive rats treated with enalapril (2K-1C + E, 20 mg/kg/day), losartan (2K-1C + L, 30 mg/kg/day), enalapril plus losartan (2K-1C + E + L, 30 and 20 mg/kg/day, respectively), or vehicle (2K-1C) and vehicle-treated control rats (2K) containing equal amounts of membrane proteins (20 μg for podocin and nephrin and 5 μg for actin) were subjected to SDS-PAGE, after which the proteins were transferred to a PVDF membrane and incubated with primary antibodies. Actin was used as a loading control. Representative immunoblots of electrophoresed renal membrane proteins and graphical representation of the relative expression of **(A)** podocin and **(B)** nephrin. The values represent individual measurements and the means ± SEM. *P < 0.05 and ****P < 0.0001 vs. 2K; ^#^*P* < 0.05, ^##^*P* < 0.01, and ^####^*P* < 0.0001 vs. 2K-1C; ^&&^*P* < 0.01 vs. 2K-1C + E; ^$$^*P* < 0.01 vs. 2K-1C + L.

Given that enalapril and losartan alone or combined seemed to reduce glomerular proteinuria, we next sought to examine whether RAS inhibition could modulate nephrin and podocin expression (Figure 3). As noted, there was a ∼55% reduction in podocin expression in 2K-1C rats in comparison with 2K rats (*P* < 0.0001) (Figure 3A). Losartan and enalapril in monotherapy partially prevented podocin downregulation. The treatment of 2K-1C rats with enalapril combined with losartan was superior to monotherapy (*P* < 0.05) and normalized podocin expression levels to those of 2K rats. The expression of nephrin remained unchanged among the groups (Figure 3B).

### Effect of Dual Versus Single RAS Blockade on the Expression of Selected Key Components of the Proximal Tubular Endocytic Machinery

**FIGURE 4:**
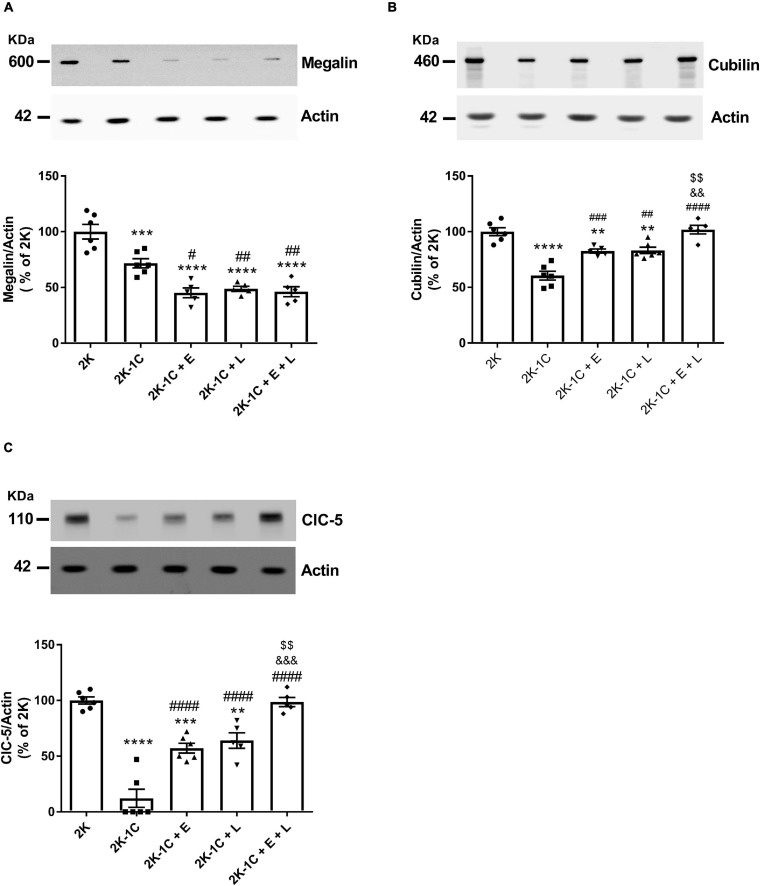
Effect of dual versus single RAS blockade on the expression of selected key components of the proximal tubular endocytic machinery in the non-clipped kidney of renovascular hypertensive rats. Samples isolated from the non-clipped kidney of renovascular-hypertensive rats treated with enalapril (2K-1C + E, 20 mg/kg/day), losartan (2K-1C + L, 30 mg/kg/day), enalapril plus losartan (2K-1C + E + L, 30 and 20 mg/kg/day, respectively), or vehicle (2K-1C) and vehicle-treated control rats (2K) containing equal amounts of membrane proteins (10 μg for megalin, 20 μg for cubilin and ClC-5 nephrin and 5 μg for actin) were subjected to SDS-PAGE, after which the proteins were transferred to a PVDF membrane and incubated with primary antibodies. Actin was used as a loading control. Representative immunoblots of electrophoresed renal membrane proteins and graphical representation of the relative expression of **(A)** megalin, **(B)** cubilin, and **(C)** ClC-5. The values represent individual measurements and the means ± SEM. **P < 0.01, ***P < 0.001, and ****P < 0.0001 vs. 2K; ^#^*P* < 0.05, ^##^*P* < 0.01, ^###^*P* < 0.001, and ^####^*P* < 0.0001 vs. 2K-1C; ^&&^*P* < 0.01 and ^&&&^*P* < 0.001 vs. 2K-1C + E; ^$$^*P* < 0.01 vs. 2K-1C + L.

Enalapril and losartan alone or combined also appeared to reduce tubular proteinuria, suggesting that RAS inhibition may upregulate the expression of components of the proximal tubular endocytic machinery. There was a 30% reduction in megalin expression in 2K-1C rats than 2K rats (Figure 4A). Treatment of 2K-1C rats with enalapril, losartan, or enalapril with losartan combined further and similarly reduced megalin expression (Figure 4A). On the other hand, the reduction in cubilin expression observed in 2K-1C rats compared to 2K rats (60 ± 4 vs. 100 ± 4%, *P* < 0.0001) was similarly attenuated by the treatment with enalapril and losartan and restored to control 2K levels when both drugs were combined (Figure 4B). Interestingly, 2K-1C rats showed a marked reduction (12 ± 5 vs. 100 ± 3%, *P* < 0.0001) in the relative expression of CIC-5 compared to 2K rats (*P* < 0.0001) (Figure 4C). The therapy with either enalapril or losartan alone attenuated the decrease in ClC-5 protein abundance. In contrast, treatment of 2K-1C rats with the combined therapy completely restored ClC-5 renal expression to the levels of 2K rats (Figure 4C).

### Effect of Dual Versus Single RAS Blockade on Renal Fibrosis

**FIGURE 5:**
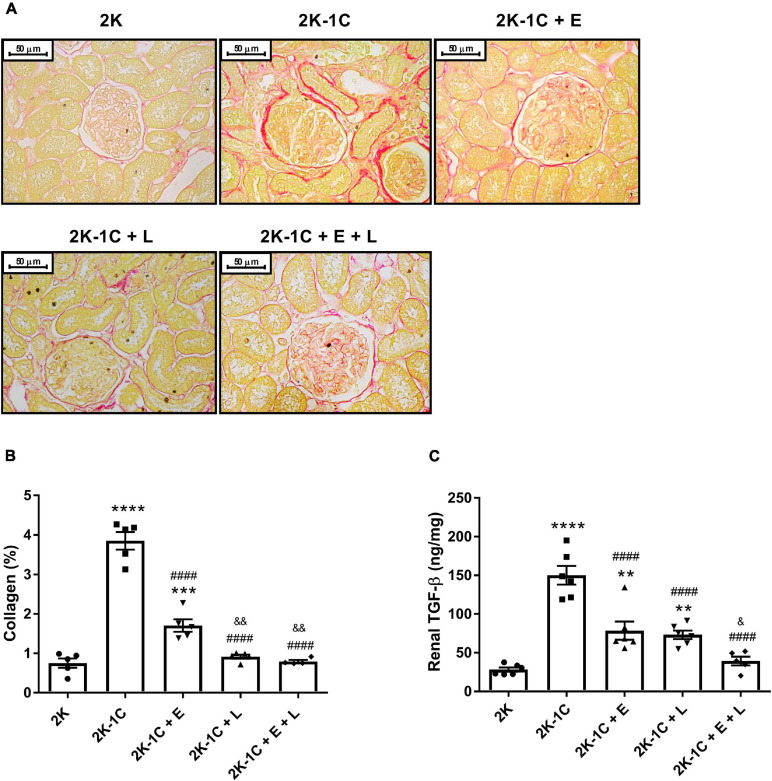
Effect of dual versus single RAS blockade on fibrosis of the non-clipped kidney of renovascular hypertensive rats. **(A)** Representative images of Picrosirius Red staining non-clipped kidney sections (400 × magnification) from renovascular-hypertensive rats treated with enalapril (2K-1C + E, 20 mg/kg/day), losartan (2K-1C + L, 30 mg/kg/day), enalapril plus losartan (2K-1C + E + L, 30 and 20 mg/kg/day, respectively), or vehicle (2K-1C) and vehicle-treated control rats (2K). The scale bar shows 50 μm. **(B)** Quantification of Picrossiuris Red staining kidney sections. The levels of non-clipped kidney collagen were expressed as percent of the total cross-section area. **(C)** The concentration of TGF-β was measured in non-clipped kidneys homogenate by ELISA. The values represent individual measurements and the means ± SEM. **P < 0.01, ***P < 0.001, and ****P < 0.0001 vs. 2K; ^####^*P* < 0.0001 vs. 2K-1C; ^&^*P* < 0.05 and ^&&^*P* < 0.01 vs. 2K-1C + E.

Figure 5A shows a representative histological image of non-clipped kidney tissue sections from each group of rats stained with Picrossirius Red. The percentage of collagen deposition was significantly higher in the non-clipped kidney of 2K-1C than 2K rats (Figure 5B). The degree of renal fibrosis in vehicle-treated 2K-1C rats was accompanied by a higher content of TGF-β (150 ± 12 ng/g vs. 28 ± 7 ng/g in 2K rats, *P* < 0.0001) (Figure 5C). The treatment of 2K-1C rats with RAS inhibitors effectively reduced collagen accumulation. An additional effect was observed for losartan or its combination with enalapril in comparison with enalapril alone (*P* < 0.005) (Figure 5B). When compared with the 2K-1C group, the levels of TGF-β was also reduced (*P* < 0.0001) by the treatment with enalapril (78 ± 12 ng/g), losartan (74 ± 5 ng/g), or by the combination enalapril/losartan (39 ± 6 ng/g). A superior reduction of TGF-β levels was observed in 2K-1C rats treated with losartan or by its combination with enalapril when compared to monotherapy with enalapril (*P* < 0.05) (Figure 5C).

### Effect of Dual Versus Single RAS Blockade on Potassium Homeostasis

**FIGURE 6:**
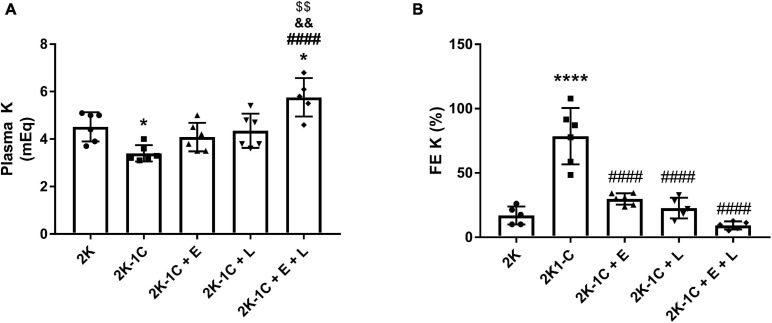
Dual RAS blockade, but not losartan or enalapril alone, induces hyperkalemia in renovascular hypertensive rats. **(A)** The plasma potassium concentration (Plasma K^+^) and **(B)** the Fractional excretion of potassium (FE K^+^) were determined in normotensive (2K) and renal hypertensive (2K-1C) rats treated for 14 days with enalapril (2K-1C + E, 20 mg/kg/day), losartan (2K-1C + L, 30 mg/kg/day), enalapril plus losartan (2K-1C + E + L, 30 and 20 mg/kg/day, respectively), or vehicle (2K and 2K-1C, water: 1 ml/kg). The values represent individual measurements and the means ± SEM. *P < 0.05 and ****P < 0.0001 vs. 2K, ^####^*P* < 0.0001 vs. 2K-1C; ^&&^*P* < 0.01 vs. 2K-1C + E; ^$$^*P* < 0.01 vs. 2K-1C + L.

Figure 6 shows that 2K-1C hypertensive rats had a lower concentration of plasma potassium compared to 2K rats (3.4 ± 0.1 vs. 4.5 ± 0.2 mEq, *P* < 0.05) (Figure 6A), which is associated with an increase in the fractional potassium excretion (78 ± 9 vs. 17 ± 3%, *P* < 0.0001) (Figure 6B). No differences were found in plasma potassium concentration between 2K-1C rats treated with either enalapril or losartan (4.1 ± 0.2 and 4.3 ± 0.3 mEq, respectively) and 2K normotensive rats (4.5 ± 0.2 mEq) (Figure 6A). Similarly, no differences were found in the fractional potassium excretion (Figure 6B) between 2K-1C rats treated with enalapril or losartan and 2K rats. Conversely, when 2K-1C rats were treated with the combination of enalapril and losartan, their plasma potassium concentration raised to a level higher than 2K rats (5.8 ± 0.4 vs. 4.5 ± 0.2 mEq, *P* < 0.05) (Figure 6A). This hyperkalemic status in 2K-1C rats treated with the combined therapy was accompanied by a non-significant decrease in fractional excretion of potassium (9 ± 1 vs. 17 ± 3% in 2K rats) (Figure 6B). Lower plasma potassium concentration in 2K-1C rats was accompanied by a higher plasma concentration of aldosterone (Table 1). The plasma aldosterone concentration did not vary significantly between 2K rats and 2K-1C rats treated with enalapril or losartan as monotherapy or the combination of enalapril and losartan (Table 1).

### Potential Role of ENaC in Mediating Potassium Disturbances in Response to Dual RAS Blockade

**FIGURE 7:**
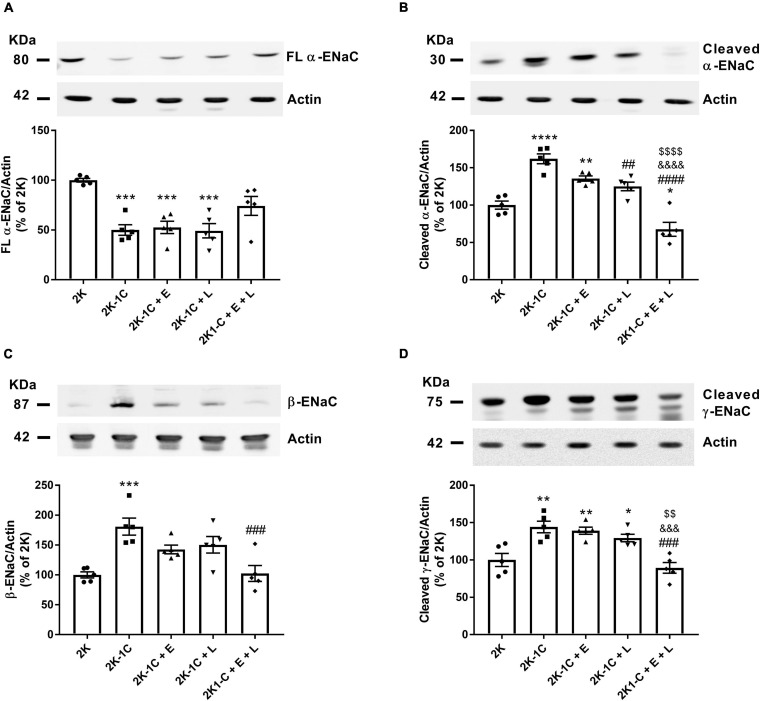
Effect of dual versus single RAS blockade on the expression of total and cleaved ENaC subunits in the non-clipped kidney of renovascular hypertensive rats. Samples isolated from the non-clipped kidney of renovascular-hypertensive rats treated with enalapril (2K-1C + E, 20 mg/kg/day), losartan (2K-1C + L, 30 mg/kg/day), enalapril plus losartan (2K-1C + E + L, 30 and 20 mg/kg/day, respectively), or vehicle (2K-1C) and vehicle-treated control rats (2K) containing equal amounts of membrane proteins (75 μg for α-ENaC, β-ENaC, and γ-ENaC and 20 μg for actin) were subjected to SDS-PAGE, after which the proteins were transferred to a PVDF membrane and incubated with primary antibodies. Actin was used as a loading control. Representative immunoblots of electrophoresed renal membrane proteins and graphical representation of the relative expression of **(A)** Full-length (FL) α-ENaC, **(B)** cleaved α-ENaC, **(C)** β-ENaC, **(D)** cleaved γ-ENaC. The values represent individual measurements and the means ± SEM. *P < 0.05, **P < 0.01, ***P < 0.001 and ****P < 0.0001 vs. 2K; ^##^*P* < 0.01, ^###^*P* < 0.001, and ^####^*P* < 0.0001 vs. 2K-1C; ^&&&^*P* < 0.001 and ^&&&&^*P* < 0.0001 vs. 2K-1C + E; ^$$^*P* < 0.01 and ^$$$$^*P* < 0.0001 vs. 2K-1C + L.

The epithelial Na^+^ channel (ENaC) mediates the passive sodium entry across the apical membrane of the principal cells in the late distal tubule and collecting ducts and consists of a heterotrimer with a single α, β, and γ subunit (Bhalla and Hallows, 2008). Proteolytic processing of α and γ subunits are markers for apical membrane ENaC activation (Kleyman et al., 2009; Palmer et al., 2012). When the apical membrane potential becomes depolarized by increased sodium entry through activated ENaC, potassium secretion across this membrane increases, and potassium is lost to the urine (Bubien, 2010). We then tested the hypothesis that the hyperkalemia induced by double RAS blockade in 2K-1C rats could be associated with ENaC subunits’ downregulation (Figure 7). As seen in Figure 7A, full-length α-ENaC expression was ∼50% reduced in vehicle-treated 2K-1C rats and 2K-1C rats treated with enalapril or losartan compared to 2K animals. No difference was found in full-length α-ENaC expression in the non-clipped kidneys of 2K-1C rats treated with the combined therapy and 2K rats (Figure 7A). In contrast, the abundance of the active cleaved α-ENaC levels was 62% increased in vehicle-treated 2K-1C, and it was also higher in 2K-1C rats treated with enalapril than in 2K rats (Figure 7B). The combined enalapril and losartan treatment considerably reduced the abundance of the active form of α-ENaC compared with vehicle-treated 2K-1C and 2K-1C rats treated with either enalapril or losartan alone (Figure 7B). Indeed, dual RAS blockade lowered the abundance of cleaved α-ENaC to levels lower than those of 2K control rats (38 ± 9 vs. 100 ± 5%, *P* < 0.05) (Figure 7B). The β-ENaC subunit was ∼75% more expressed in the non-clipped kidney of vehicle-treated 2K-1C rats than in 2K rats (*P* < 0.05) (Figure 7C). The combined therapy of enalapril and losartan but not the monotherapy effectively reduced the β-ENaC subunit expression in 2K-1C rats (Figure 7C). The expression of the active cleaved form of γ-ENaC was increased in the non-clipped kidney of vehicle-treated 2K-1C rats compared to 2K rats (144 ± 8 vs. 100 ± 9%, *P* < 0.01). Only the combined therapy could restore the cleaved γ-ENaC abundance to the levels of 2K rats (Figure 7D).

## Discussion

This study explored the potential molecular mechanisms underlying the antiproteinuric and hyperkalemic effects of dual RAS blockade in renovascular hypertensive rats. We found that the better management of proteinuria of 2K-1C rats in response to the combination of enalapril with losartan was associated with higher protein abundance of podocin and cubilin in the non-clipped kidney relative to either enalapril- or losartan -treated 2K-1C rats. Additionally, we showed that higher plasma potassium concentration in 2K-1C rats treated with the combination of enalapril and losartan exhibited downregulation of the cleaved/activated forms of α-ENaC and γ-ENaC in the non-clipped kidney, suggesting that posttranslational modification of these ENaC subunits in the collecting duct may contribute to dual RAS blockade-induced hyperkalemia.

Hyperactivation of the renal RAS limits the kidney’s ability to maintain sodium balance at normal arterial pressures and contributes to hypertension development (Crowley et al., 2006; Navar et al., 2011). Renovascular hypertension in the 2K-1C model is initially induced by renin secretion stimulation caused by decreased renal perfusion, leading to increased systemic Ang II concentration. Interestingly, despite circulating Ang II levels return to normal at the hypertensive state, hypertension becomes stable as renal RAS is maintained abnormally overactive (Navar et al., 1998). We found that the Ang II content in the non-clipped kidney of renovascular hypertensive 2K-1C rats was markedly elevated. We also observed that either enalapril or losartan treatment reduced Ang II levels in the non-clipped kidney of 2K-1C, potentially favoring the natriuretic ability in these animals and setting an intermediate hypertensive state. However, the dual RAS blockade that blunts both AT1R activation and Ang II production by ACE was capable of restoring the renal concentration of Ang II to normotensive rats’ levels and, therefore, the ability of the kidneys to maintain sodium balance. As a result, the combined therapy of enalapril and losartan was more effective than either drug alone in reducing BP in 2K-1C.

Ang II can substantially modulate proteinuria and albuminuria through effects on BP, and independently thereof, through changes of intraglomerular hemodynamics and modulation of critical components of the glomerular filtration barrier and the apical endocytic machinery in the renal proximal tubule (Langham et al., 2004; Long et al., 2004; Mann and Böhm, 2015; Aroor et al., 2016). The relative contribution of elevated BP versus Ang II on the induction of renal injury has been previously quantified by Mori and Cowley (2004) in the experimental model of Ang II-induced hypertension. Using servo-controlled kidneys that eliminated the hypertensive environment from one kidney, these authors observed that kidney injury markers were mainly induced by hypertension. Nevertheless, the servo-controlled kidney still has higher levels of injury markers than the sham group, which supports that RAS overactivation can affect kidney integrity by a pressure-independent mechanism and, plausibly, by direct local activation of Ang II production and signaling. As expected, 2K-1C rats presented overt proteinuria and albuminuria, which were attenuated by monotherapy with either enalapril and losartan and restored to control levels normotensive rats by the combined treatment. Considering previous evidence, such normalization would involve not only the control of BP by the combination of enalapril and losartan but also its underlying suppression of overactive local RAS.

Podocin and nephrin are two essential components of the glomerular filtration barrier, whose integrity is responsible for restricting the loss of high-molecular proteins in the urine. We observed that, whereas nephrin expression is not changed in 2K-1C rats, the protein abundance of podocin is significantly downregulated. It is known that Ang II and mechanical forces induce autophagy and apoptosis in podocyte culture (Yadav et al., 2010; Huang et al., 2012; Zhao et al., 2018), which could cause a generalized loss of both nephrin and podocin expression. At the same time, Ang II treatment can regulate nephrin and podocin transcription. Ang II treatment decreases podocin mRNA levels *in vitro* (Yang et al., 2017), which would favor podocin expression reduction in Ang II-dependent hypertensive settings. Accordingly, ACEi and ARB treatment partially reverted, and combined treatment completely reverted downregulation of podocin expression, which is in line with the efficiency of BP control and normalization of renal RAS obtained by such therapies. On the other hand, Ang II regulation of nephrin expression seems more complex. Rats receiving Ang II pressor doses of Ang II during 10–14 days showed increased nephrin expression at mRNA (Langham et al., 2004; Jia et al., 2008) and protein levels (Jia et al., 2008). However, after 28 days of Ang II infusion, nephrin mRNA and protein abundance start to decline in comparison with basal levels, and such reduction was associated with the appearance of apoptosis in podocytes. The absence of differences in nephrin expression in 2K-1C rats in comparison with sham rats may reflect the balance between these opposite regulatory processes, i.e., induction of nephrin transcription by increases in renal Ang II levels and downregulation of podocyte proteins due to apoptosis induced, directly or indirectly, by chronic Ang II stimulus.

Renovascular hypertensive 2K-1C rats also exhibited increased low-molecular-weight proteins in the urine, suggesting abnormal handling of filtrated smaller proteins by the renal tubule. Endocytic receptors in the proximal tubule (megalin and cubilin) are involved in handling, internalizing filtrated proteins, and avoiding their urine loss. In line with the analysis of the pattern of proteinuria, cubilin and megalin expressions in the cortical membranes of the non-clipped kidney were reduced in 2K-1C rats. Such reductions might be partly caused by tubular injury observed in Ang II-dependent hypertensive models (Mori and Cowley, 2004). Nevertheless, Ang II-dependent local signaling and fine control of protein expression are probably also involved. Aroor and colleagues (Aroor et al., 2016) observed that decreased megalin expression in rats receiving Ang II infusion is associated with increased kidney DPP4 activity since DPP4 inhibition partially restored megalin expression in these animals. Moreover, such regulation of megalin expression was also observed in proximal tubule cells *in vitro*. Another critical molecular mechanism regulating the expression of endocytic receptors in the proximal tubule is TGF-β (Gekle et al., 2003), a factor that we found to be upregulated in the non-clipped kidney of 2K-1C rats. Proximal tubule-like OKP cell line treated with TGF-β have lower levels of both megalin and cubilin and lower protein endocytosis capacity. This regulation seems dependent on the induction of the transcription factors Smad2/3.

Dual RAS blockade reverted cubilin downregulation, which is expected by attenuation of kidney injury, TGF-β levels, and local RAS signaling in treated rats. Unexpectedly, RAS inhibitors further decreased megalin expression in the non-clipped kidney of 2K-1C treated rats. Such downregulation of megalin may be associated with the glycosuria presented in hypertensive rats treated with enalapril and/or losartan. Notably, cumulative evidence demonstrates the existence of an interplay between Ang II and sodium-glucose cotransporter 2 (SGLT2)-mediated glucose uptake in the renal proximal tubule (Silva Dos Santos et al., 2020). Accordingly, Bautista and colleagues (Bautista et al., 2004) have shown that renovascular hypertensive rats display higher activity in renal brush border membrane vesicles, an effect that was inhibited by treatment with ACEi or ARB. These findings suggest that the glycosuria observed in 2K-1C treated with RAS inhibitors might be induced by SGLT2 downregulation. Interestingly, there is evidence that SGLT2 modulates megalin cell surface expression in the renal proximal tubule. Using an animal model of diabetic nephropathy, Otomo and colleagues (Otomo et al., 2020) observed that SGLT2 inhibition suppressed O-GlcNAcylation of megalin, contributing to megalin internalization. Regardless of the mechanism responsible for further reducing megalin in 2K-1C treated with RAS inhibitors either in monotherapy or combination, the normalization of cubilin and podocin expression by dual RAS blockade seems sufficient to abrogate the overt proteinuria observed in 2K-1C untreated rats.

In the present study, we observed for the first time that another critical component of the apical endocytic machinery in the renal proximal tubule, the ClC-5, is remarkably downregulated in the non-clipped kidney of renovascular hypertensive 2K-1C rats. Cultures of proximal tubule cells from ClC-5 knockout mice showed that ClC-5 is critical for proper acidification of early endosomes, thus explaining why Dent’s disease, caused by ClC-5 mutations, is characterized by low-molecular-weight proteinuria (Günther et al., 1998). It was reported previously that microalbuminuric SHR displays reduced renal cortical ClC-5 protein abundance compared to WKY rats. This reduction was attenuated by ACEi treatment, suggesting that Ang II downregulates ClC-5 expression by a pressure mechanism and/or by a local RAS signaling (Tanaka and Nakaki, 2004). Accordingly, we observed that renal cortical expression of ClC-5 was reverted, at least in part, by ACEi and ARB treatment and normalized by dual RAS blockade.

We also assessed how ACEi and ARB treatment alone or in combination impacts fibrosis in the non-clipped kidney of 2K-1C rats. TGF-β is recognized as a vital mediator in kidney fibrosis. Mechanical forces are known to induce TGF-β signaling and neutralization of TGF-β with antibodies abrogates the increase in collagen synthesis in vascular smooth muscle cells (O’Callaghan and Williams, 2000). Therefore, upregulation of TGF-β and collagen deposition observed in the non-clipped kidney of 2K-1C rats might be at least partially attributed to the hypertensive state, and these mechanisms are blunted by ACEi and ARB treatments that reduce BP levels. Moreover, Ang II is involved in direct signaling pathways triggering TGF-β secretion (Wolf, 2006) in such a way that ACEi and ARBs might also attenuate TGF-β signaling by a pressure-independent mechanism. Interestingly, collagen deposition differs between enalapril-treated and losartan-treated 2K-1C rats, despite similar TGF-β levels, suggesting that other molecular factors may contribute to kidney fibrosis under these conditions. Losartan-mediated blockade of AT1R might be more efficient in normalizing collagen deposition since losartan treatment not only decreases renal Ang II synthesis, as ACEi treatment does, but also directly blocks AT1R signaling. It is known that AT2R stimulation induces a decrease in collagen synthesis (Danyel et al., 2013), and losartan-treated kidneys might switch from an Ang II signaling to an AT2R-dependent pathway, thus further reducing collagen deposition.

Even though dual RAS therapy is superior to monotherapy for BP control and proteinuria, such superiority does not translate into improvements in long-term clinical outcomes. Moreover, dual blockade of the RAS is associated with a higher prevalence of hyperkalemia in patients with hypertension. As potassium net secretion is defined by modulation of ion transport at the distal nephron (Malnic et al., 1964), we assessed the effect of dual RAS blockade on the modulation of a critical channel that indirectly favors potassium secretion, the epithelial sodium channel (ENaC). Indeed, the inhibition of ENaC impairs renal potassium secretion and causes hyperkalemia, whereas the stimulation of ENaC increases renal potassium secretion and leads to hypokalemia. Accordingly, we observed that 2K-1C rats presented lower levels of plasma potassium associated with higher fractional excretion of potassium and increased expression of the active cleaved forms of α-ENaC and γ-ENaC and upregulation of β-ENaC. On the other hand, 2K-1C rats treated with either ACEi or ARB alone had lower markers of ENaC activation compared to vehicle-treated 2K-1C rats, and K^+^ excretion was attenuated. The combined therapy of ACEi and ARB normalized the abundance of β-ENaC and cleaved γ-ENaC in the non-clipped kidney of 2K-1C rats. Notably, dual RAS blockade decreased the renal cortical content of cleaved α-ENaC to levels that were even lower than those of 2K normotensive rats, which may explain, at least in part, the presence of hyperkalemia.

It is known that both α- and γ-ENaC are activated by double cleavage of N-terminal sites and consequent release of auto-inhibitory peptides (Carattino et al., 2008; Passero et al., 2010). α-ENaC is cleaved twice by furin, an intracellular protease that resides in the trans-Golgi network (TGN) (Carattino et al., 2008). On the other hand, γ-ENaC is cleaved once by furin and then cleaved at another site by other collecting duct membrane proteases (Frindt et al., 2016). It is also known that ENaC in rat kidneys can bypass TGN trafficking and, thus, furin cleavage step, resulting in a heterogeneous population of cleaved and non-cleaved ENaC channels plasma membrane. Evidence suggests that ENaC trafficking through TGN is regulated positively by a low-salt diet, implicating the involvement of RAS (Frindt et al., 2016). In this regard, Dooley and colleagues (Dooley et al., 2013) observed that aldosterone treatment in collecting duct cells induces a protein complex at TGN that is essential for ENaC induced exocytosis. As far as we know, it is not clear whether Ang II can also directly modulate similar regulatory processes at TGN. Our observations that α-ENaC cleavage is upregulated in hypertensive 2K-1C rats and then downregulated by combined treatment are in line with these previous observations. These findings suggest that Ang II signaling upregulates, either directly or indirectly, the cleavage of α-ENaC and, consequently, the activity of the channel.

Moreover, the β-ENaC expression is also upregulated in some hypertension models, such as SHR and Dahl salt-sensitive rats (Haloui et al., 2013; Pavlov et al., 2013). Although previous studies have not shown evidence for direct stimulation of β-ENaC expression by Ang II or aldosterone (Masilamani et al., 1999; Loffing et al., 2001; Beutler et al., 2003), recent work demonstrated that a non-pressor dose of Ang II is able to upregulate β-ENaC protein expression, even in mineralocorticoid receptor-knockout mice (Wu et al., 2020). This is in line with our results that showed higher β-ENaC expression in cortical membranes of 2K-1C rats, partial normalization by ACEi or ARB treatment, and normalization to control levels in combined therapy.

Other mechanisms for modulation of potassium excretion during ACEi and ARB therapy may not be ruled out. Indeed, ACEi treatment seems to considerably reduce the fractional excretion of potassium compared to vehicle-treated 2K-1C rats, despite modest changes in ENaC regulation. It is known that BK channels in the distal nephron can be activated by flow, thus increasing potassium secretion (Liu et al., 2009). We have observed that 2K-1C rats had significantly increased urinary flow, and that was attenuated by ACEi and ARB treatments. Such diuresis reduction might also be important to attenuate potassium excretion in ACEi- and ARB-treated 2K-1C rats. Lee and colleagues (Lee et al., 2001) have observed that the increase in urinary flow observed in 2K-1C rats was associated with a decreased expression of AQP2 in the clipped kidney, especially in the outer medulla region. Interestingly, the downregulation of AQP2 was reverted when the kidney was unclipped for 24 h. They also observed that clipped kidney medulla is less responsive to vasopressin, an effect that was also reverted by 1-day unclipping. These observations suggest that decreased kidney perfusion might blunt AVP response and, then, antidiuretic mechanisms. Accordingly, collecting duct cells chronically treated with activators of AMPK, a protein that is activated by hypoxia, reduces the cell ability to AQP2 upregulation in response to forskolin (Al-Bataineh et al., 2016). Dual RAS blockade may ameliorate renal perfusion, thus improving antidiuresis. It is known that cortical and medullary perfusion in 2K-1C is decreased even in unclipped kidneys compared to sham rat kidneys, despite a higher BP (Wickman et al., 2001). This event might be associated with Ang II-induced hemodynamic changes in afferent and efferent arterioles. Accordingly, it is reported that peritubular capillary perfusion is reduced by Ang II administration in rats and patients (Nangaku and Fujita, 2008). Reducing renal RAS status by ACEi and ARB therapy attenuates vasoconstriction in glomeruli, thus improving perfusion to downstream vascular beds.

In conclusion, we demonstrate that dual RAS blockade may ameliorate proteinuria by preventing downregulation of components of the glomerular filtration barrier and the apical endocytic machinery in the renal proximal tubule. While dual RAS blockade normalized the expression of podocin, cubilin, and ClC-5, the single blockade of the RAS only attenuated the Ang II and/or BP-induced downregulation of these proteins may explain the superior antiproteinuric effect of dual vs. single RAS blockade in renovascular hypertensive rats. Additionally, we show that dual vs. single RAS blockade differentially impacts renal ENaC modulation at both translational and posttranslational levels. Moreover, we observed that dual RAS blockade-induced hyperkalemia is associated with exacerbated downregulation of the active cleaved form of α-ENaC.

## Data Availability Statement

The raw data supporting the conclusions of this article will be made available by the authors, without undue reservation.

## Ethics Statement

The animal study was reviewed and approved by the Institutional Animal Care and Use Committee of the University of São Paulo, Ribeirão Preto, SP, Brazil.

## Author Contributions

JC, LB, and AG designed the study. JC, KB, LS, TS, and FM carried out the experiments. JC, KB, LS, JP, and FM analyzed the data. JC, JP, and AG wrote the manuscript. JC and LS prepared the figures. All authors contributed to the article and approved the submitted version.

## Conflict of Interest

The authors declare that the research was conducted in the absence of any commercial or financial relationships that could be construed as a potential conflict of interest.
